# *trans*-Bis(2-benzoyl­benzoato-κ*O* ^1^)bis­(ethanol-κ*O*)bis­(1*H*-imidazole-κ*N* ^3^)nickel(II)

**DOI:** 10.1107/S160053680902621X

**Published:** 2009-07-11

**Authors:** Zerrin Heren, Hümeyra Paşaoğlu, M. Hakkı Yıldırım, Derya Hıra

**Affiliations:** a Department of Chemistry, Faculty of Arts and Sciences, Ondokuz Mayıs University, Kurupelit, TR-55139 Samsun, Turkey; b Department of Physics, Faculty of Arts and Sciences, Ondokuz Mayıs University, 55139 Kurupelit Samsun, Turkey

## Abstract

In the title centrosymmetric mononuclear nickel(II) complex, [Ni(C_14_H_9_O_3_)_2_(C_3_H_4_N_2_)_2_(CH_3_CH_2_OH)_2_], the central Ni^II^ ion lies on an inversion centre and is octa­hedrally coordinated. The equatorial plane is formed by two O atoms from two symmetry-related 2-benzoyl­benzoate ligands and two N atoms from two symmetry-related imidazole ligands, whereas the axial positions are occupied by two O atoms from two ethanol ligands. Intramolecular O---H...O hydrogen bonds stabilize this arrangement. The mol­ecules are linked into chains running along the *b* axis by N—H⋯O hydrogen bonds.

## Related literature

For crystal structures with 2-benzoyl­benzoate ligands, see: Diop *et al.* (2006 ▶, 2007 ▶); Foreman *et al.* (2001 ▶); Jones *et al.* (1996 ▶); Martin & Valente (1998 ▶); Prout *et al.* (1996 ▶); Song *et al.* (2005 ▶); Yıldırım *et al.* (2009 ▶). For the crystal structure of 2-benzoyl­benzoic acid, see: Lalancette *et al.* (1990 ▶). For graph-set notation, see: Bernstein *et al.* (1995 ▶).
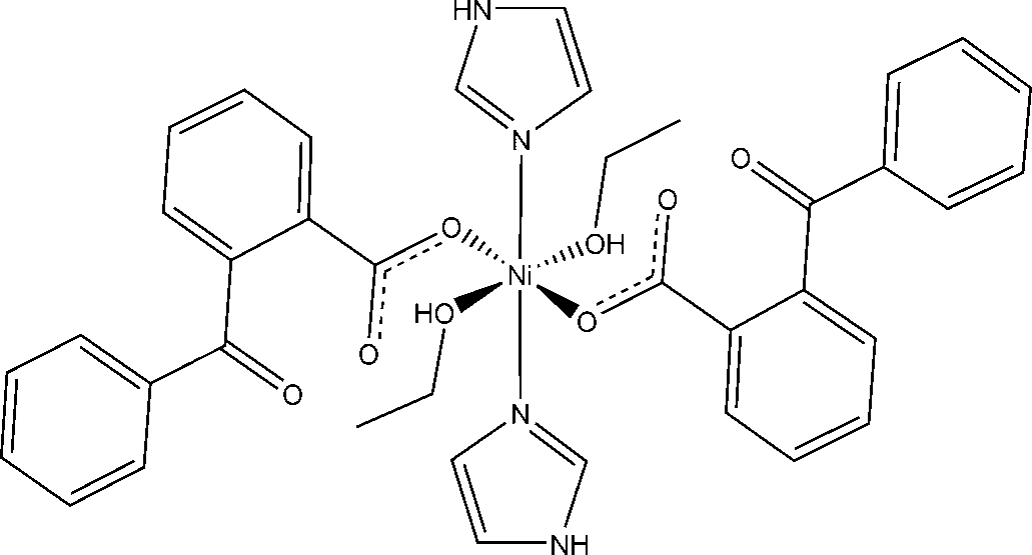


## Experimental

### 

#### Crystal data

[Ni(C_14_H_9_O_3_)_2_(C_3_H_4_N_2_)_2_(C_2_H_6_O)_2_]
*M* *_r_* = 737.43
Monoclinic, 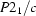

*a* = 12.8914 (8) Å
*b* = 8.3908 (5) Å
*c* = 16.4590 (11) Å
β = 90.832 (5)°
*V* = 1780.17 (19) Å^3^
*Z* = 2
Mo *K*α radiation
μ = 0.60 mm^−1^
*T* = 296 K
0.43 × 0.25 × 0.14 mm

#### Data collection

Stoe IPDSII diffractometer
Absorption correction: integration (*X-RED32*; Stoe & Cie, 2002 ▶) *T*_min_ = 0.809, *T*_max_ = 0.921
15078 measured reflections
3889 independent reflections
2704 reflections with *I* > 2σ(*I*)
*R*_int_ = 0.046

#### Refinement

*R*[*F* ^2^ > 2σ(*F* ^2^)] = 0.055
*wR*(*F* ^2^) = 0.150
*S* = 1.11
3889 reflections
235 parameters
H atoms treated by a mixture of independent and constrained refinement
Δρ_max_ = 0.31 e Å^−3^
Δρ_min_ = −0.26 e Å^−3^

### 

Data collection: *X-AREA* (Stoe & Cie, 2002 ▶); cell refinement: *X-AREA*; data reduction: *X-RED32* (Stoe & Cie, 2002 ▶); program(s) used to solve structure: *SHELXS97* (Sheldrick, 2008 ▶); program(s) used to refine structure: *SHELXL97* (Sheldrick, 2008 ▶); molecular graphics: *ORTEP-3 for Windows* (Farrugia, 1997 ▶); software used to prepare material for publication: *WinGX* (Farrugia, 1999 ▶).

## Supplementary Material

Crystal structure: contains datablocks global, I. DOI: 10.1107/S160053680902621X/ci2845sup1.cif

Structure factors: contains datablocks I. DOI: 10.1107/S160053680902621X/ci2845Isup2.hkl

Additional supplementary materials:  crystallographic information; 3D view; checkCIF report

## Figures and Tables

**Table 1:** Hydrogen-bond geometry (Å, °)

*D*—H⋯*A*	*D*—H	H⋯*A*	*D*⋯*A*	*D*—H⋯*A*
O1—H1⋯O3	0.78 (5)	1.86 (5)	2.627 (4)	170 (6)
N2—H2⋯O3^i^	0.86	2.02	2.837 (5)	159

Symmetry code: (i) 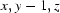.

**Fig. 1.**
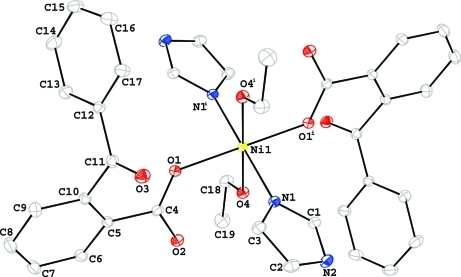
The molecular structure of the title complex, showing the atom-numbering scheme. Displacement ellipsoids are drawn at the 20% probability. Symmetry code: (i) 1 -x, 1 -y, 1 -z.

**Fig. 2.**
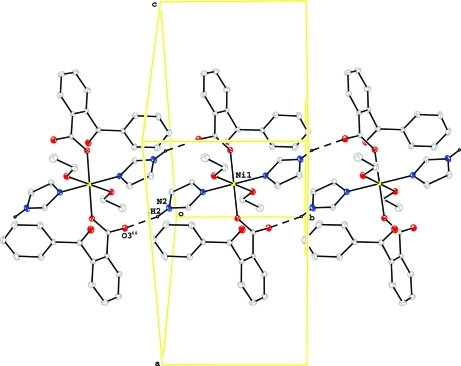
Part of the crystal structure of the title compound showing the chain of R22(16) rings along [010] generated by N—H···O hydrogen bonds (dashed lines). H atoms not involved in hydrogen bonds have been omitted for clarity.Symmetry code: (ii) x, y - 1, z

## supplementary crystallographic information

### Comment

We have previously reported the crystal structure of [Cu(2-byba)_2_(bim)_2_] (bim = benzimidazole, 2-byba = 2-benzoylbenzoate) (Yıldırım *et al.*, 2009). As an extension of this study, we now report the structure of a new nickel(II) complex with the 2-byba ligand.

In the title complex, the Ni^II^ ion lies on a centre of symmetry and has a octahedral coordination geometry formed by 2-byba, imidazole (im), ethanol ligands and their symmetry-related equivalents. All ligands are monodentate with the 2-byba coordinates through a carboxylate O atom, im coordinates through the aromatic N atom and ethanol coordinates through the O atom. Intramolecular O—H···O hydrogen bonds are observed.

The molecular packing is mainly stabilized by strong intermolecular N—H···O hydrogen bonds (Table 2 and Fig. 2). Atom N2 in the molecule at (*x*, *y*, *z*) acts as a hydrogen-bond donor, *via* H2, to atom O3 in the molecule at (*x*, *y* - 1, *z*) forming C(8) chains with *R*_2_^2^(16) rings (Bernstein *et al.*, 1995). These chains run parallel to the [010] (Fig. 2).

### Experimental

A solution of 2-benzoylbenzoate (0.45 g, 2 mmol) in ethanol (10 ml) was added to a solution of nickel acetate tetrahydrate (0.25 g, 1 mmol) in ethanol (10 ml) and the solution was stirred for 15 min at 333 K. To this solution, a solution of imidazole (0.23 g, 2 mmol) in ethanol (10 ml) was added and the resultant solution was left to evaporate slowly at room temperature. After one week, single crystals of the title complex were isolated.

### Refinement

Alcohol H atom was located in a difference Fourier map and its positional parameters were refined. The remaining H atoms were placed in calculated positions and constrained to ride on their parents atoms, with C-H = 0.93-0.97 Å, N-H = 0.86 Å and *U*_iso_(H) = 1.2*U*_eq_(C,N).

### Crystal data

**Table:**

[Ni(C_14_H_9_O_3_)_2_(C_3_H_4_N_2_)_2_(C_2_H_6_O)_2_]	*F*(000) = 772
*M**_r_* = 737.43	*D*_x_ = 1.376 Mg m^−^^3^
Monoclinic, *P*2_1_/*c*	Mo *K*α radiation, λ = 0.71073 Å
Hall symbol: -P 2ybc	Cell parameters from 15078 reflections
*a* = 12.8914 (8) Å	θ = 1.6–27.6°
*b* = 8.3908 (5) Å	µ = 0.60 mm^−^^1^
*c* = 16.4590 (11) Å	*T* = 296 K
β = 90.832 (5)°	Prism, green
*V* = 1780.17 (19) Å^3^	0.43 × 0.25 × 0.14 mm
*Z* = 2	

### Data collection

**Table:**

Stoe IPDSII diffractometer	3889 independent reflections
Radiation source: fine-focus sealed tube	2704 reflections with *I* > 2σ(*I*)
graphite	*R*_int_ = 0.046
Detector resolution: 6.67 pixels mm^-1^	θ_max_ = 27.0°, θ_min_ = 1.6°
ω scans	*h* = −16→16
Absorption correction: integration (*X-RED32*; Stoe & Cie, 2002)	*k* = −10→10
*T*_min_ = 0.809, *T*_max_ = 0.921	*l* = −20→20
15078 measured reflections	

### Refinement

**Table:**

Refinement on *F*^2^	Primary atom site location: structure-invariant direct methods
Least-squares matrix: full	Secondary atom site location: difference Fourier map
*R*[*F*^2^ > 2σ(*F*^2^)] = 0.055	Hydrogen site location: inferred from neighbouring sites
*wR*(*F*^2^) = 0.150	H atoms treated by a mixture of independent and constrained refinement
*S* = 1.11	*w* = 1/[σ^2^(*F*_o_^2^) + (0.0435*P*)^2^ + 2.752*P*] where *P* = (*F*_o_^2^ + 2*F*_c_^2^)/3
3889 reflections	(Δ/σ)_max_ = 0.004
235 parameters	Δρ_max_ = 0.31 e Å^−^^3^
0 restraints	Δρ_min_ = −0.26 e Å^−^^3^

### Special details

**Table:**

Geometry. All e.s.d.'s (except the e.s.d. in the dihedral angle between two l.s. planes) are estimated using the full covariance matrix. The cell e.s.d.'s are taken into account individually in the estimation of e.s.d.'s in distances, angles and torsion angles; correlations between e.s.d.'s in cell parameters are only used when they are defined by crystal symmetry. An approximate (isotropic) treatment of cell e.s.d.'s is used for estimating e.s.d.'s involving l.s. planes.
Refinement. Refinement of *F*^2^ against ALL reflections. The weighted *R*-factor *wR* and goodness of fit *S* are based on *F*^2^, conventional *R*-factors *R* are based on *F*, with *F* set to zero for negative *F*^2^. The threshold expression of *F*^2^ > σ(*F*^2^) is used only for calculating *R*-factors(gt) *etc*. and is not relevant to the choice of reflections for refinement. *R*-factors based on *F*^2^ are statistically about twice as large as those based on *F*, and *R*- factors based on ALL data will be even larger.

### Fractional atomic coordinates and isotropic or equivalent isotropic displacement parameters (Å^2^)

**Table:**

	*x*	*y*	*z*	*U*_iso_*/*U*_eq_	
C1	0.3658 (4)	0.5474 (6)	0.3388 (3)	0.0630 (13)	
H1A	0.3057	0.4882	0.3568	0.076*	
H1B	0.4138	0.4723	0.3151	0.076*	
C2	0.3320 (5)	0.6663 (8)	0.2749 (3)	0.0848 (18)	
H2A	0.2993	0.6112	0.2303	0.127*	
H2B	0.3915	0.7231	0.2558	0.127*	
H2C	0.2838	0.7402	0.2979	0.127*	
C3	0.5546 (4)	0.1822 (5)	0.4290 (3)	0.0525 (11)	
H3	0.6248	0.2074	0.4330	0.063*	
C4	0.4127 (4)	0.0507 (6)	0.4036 (3)	0.0623 (13)	
H4	0.3661	−0.0284	0.3879	0.075*	
C5	0.3897 (4)	0.1970 (6)	0.4334 (3)	0.0563 (11)	
H5	0.3230	0.2356	0.4416	0.068*	
C6	0.6557 (3)	0.6330 (5)	0.3827 (2)	0.0448 (9)	
C7	0.7620 (3)	0.6345 (5)	0.3466 (2)	0.0452 (9)	
C8	0.7755 (4)	0.7083 (5)	0.2710 (3)	0.0548 (11)	
H8	0.7192	0.7560	0.2448	0.066*	
C9	0.8715 (4)	0.7106 (6)	0.2352 (3)	0.0660 (13)	
H9	0.8800	0.7617	0.1856	0.079*	
C10	0.9537 (4)	0.6380 (7)	0.2724 (3)	0.0724 (15)	
H10	1.0181	0.6391	0.2477	0.087*	
C11	0.9426 (4)	0.5622 (6)	0.3471 (3)	0.0658 (13)	
H11	0.9991	0.5124	0.3720	0.079*	
C12	0.8466 (3)	0.5616 (5)	0.3841 (2)	0.0454 (9)	
C13	0.8422 (3)	0.4876 (6)	0.4681 (2)	0.0481 (9)	
C14	0.8461 (3)	0.3113 (5)	0.4742 (3)	0.0463 (10)	
C15	0.8481 (4)	0.2143 (6)	0.4068 (3)	0.0616 (12)	
H15	0.8446	0.2587	0.3551	0.074*	
C16	0.8554 (5)	0.0506 (6)	0.4159 (4)	0.0829 (17)	
H16	0.8574	−0.0150	0.3704	0.100*	
C17	0.8597 (4)	−0.0149 (7)	0.4934 (4)	0.0815 (16)	
H17	0.8650	−0.1249	0.4993	0.098*	
C18	0.8564 (5)	0.0769 (7)	0.5591 (4)	0.0770 (16)	
H18	0.8585	0.0306	0.6104	0.092*	
C19	0.8498 (4)	0.2423 (6)	0.5516 (3)	0.0613 (12)	
H19	0.8479	0.3062	0.5978	0.074*	
N1	0.4796 (3)	0.2796 (4)	0.44961 (19)	0.0431 (8)	
N2	0.5184 (3)	0.0434 (4)	0.4016 (2)	0.0601 (10)	
H2	0.5548	−0.0362	0.3857	0.072*	
Ni1	0.5000	0.5000	0.5000	0.0385 (2)	
O1	0.4142 (2)	0.6220 (4)	0.40705 (17)	0.0489 (7)	
O2	0.6371 (2)	0.5278 (3)	0.43480 (16)	0.0462 (7)	
O3	0.5923 (2)	0.7370 (4)	0.35828 (19)	0.0566 (8)	
O4	0.8429 (3)	0.5724 (4)	0.52801 (18)	0.0585 (8)	
H1	0.465 (4)	0.665 (7)	0.395 (3)	0.070*	

### Atomic displacement parameters (Å^2^)

**Table:**

	*U*^11^	*U*^22^	*U*^33^	*U*^12^	*U*^13^	*U*^23^
C1	0.080 (3)	0.056 (3)	0.052 (3)	−0.004 (2)	−0.011 (2)	−0.007 (2)
C2	0.106 (5)	0.099 (5)	0.049 (3)	0.009 (4)	−0.017 (3)	0.005 (3)
C3	0.062 (3)	0.041 (2)	0.054 (2)	−0.002 (2)	0.007 (2)	−0.003 (2)
C4	0.071 (3)	0.046 (3)	0.070 (3)	−0.008 (2)	−0.009 (2)	−0.009 (2)
C5	0.058 (3)	0.050 (3)	0.061 (3)	−0.001 (2)	−0.003 (2)	−0.007 (2)
C6	0.053 (2)	0.036 (2)	0.045 (2)	0.0040 (19)	0.0011 (18)	−0.0035 (18)
C7	0.055 (2)	0.040 (2)	0.041 (2)	−0.0023 (19)	0.0071 (18)	−0.0004 (17)
C8	0.067 (3)	0.051 (3)	0.047 (2)	−0.001 (2)	0.006 (2)	0.004 (2)
C9	0.075 (3)	0.070 (3)	0.054 (3)	−0.010 (3)	0.017 (2)	0.008 (2)
C10	0.067 (3)	0.083 (4)	0.068 (3)	−0.010 (3)	0.025 (3)	0.001 (3)
C11	0.056 (3)	0.069 (3)	0.073 (3)	0.005 (2)	0.014 (2)	0.004 (3)
C12	0.047 (2)	0.042 (2)	0.047 (2)	−0.0058 (18)	0.0040 (18)	−0.0009 (18)
C13	0.044 (2)	0.049 (2)	0.051 (2)	−0.001 (2)	0.0021 (16)	−0.002 (2)
C14	0.042 (2)	0.041 (2)	0.056 (2)	−0.0002 (18)	0.0046 (18)	0.0022 (19)
C15	0.071 (3)	0.052 (3)	0.062 (3)	−0.003 (2)	0.007 (2)	−0.006 (2)
C16	0.096 (4)	0.045 (3)	0.108 (5)	0.001 (3)	0.017 (4)	−0.020 (3)
C17	0.081 (4)	0.044 (3)	0.120 (5)	0.000 (3)	0.016 (3)	0.015 (3)
C18	0.090 (4)	0.059 (3)	0.082 (4)	−0.001 (3)	0.005 (3)	0.022 (3)
C19	0.071 (3)	0.051 (3)	0.062 (3)	−0.004 (2)	0.002 (2)	0.006 (2)
N1	0.0491 (19)	0.0369 (18)	0.0434 (17)	−0.0001 (16)	0.0029 (15)	−0.0017 (14)
N2	0.078 (3)	0.042 (2)	0.061 (2)	0.0093 (19)	0.0084 (19)	−0.0090 (17)
Ni1	0.0424 (4)	0.0347 (4)	0.0384 (3)	0.0010 (4)	0.0027 (3)	−0.0022 (3)
O1	0.0534 (18)	0.0486 (19)	0.0446 (15)	0.0003 (14)	−0.0038 (13)	−0.0035 (13)
O2	0.0521 (16)	0.0393 (17)	0.0474 (14)	0.0011 (13)	0.0080 (12)	0.0042 (12)
O3	0.0580 (19)	0.0476 (18)	0.0644 (18)	0.0100 (15)	0.0056 (15)	0.0074 (15)
O4	0.078 (2)	0.0487 (18)	0.0485 (17)	0.0015 (16)	−0.0019 (15)	−0.0077 (14)

### Geometric parameters (Å, °)

**Table:**

C1—O1	1.423 (5)	C10—H10	0.93
C1—C2	1.509 (7)	C11—C12	1.387 (6)
C1—H1A	0.97	C11—H11	0.93
C1—H1B	0.97	C12—C13	1.517 (6)
C2—H2A	0.96	C13—O4	1.216 (5)
C2—H2B	0.96	C13—C14	1.483 (6)
C2—H2C	0.96	C14—C15	1.377 (6)
C3—N1	1.315 (5)	C14—C19	1.400 (6)
C3—N2	1.331 (5)	C15—C16	1.384 (7)
C3—H3	0.93	C15—H15	0.93
C4—C5	1.356 (6)	C16—C17	1.389 (8)
C4—N2	1.365 (6)	C16—H16	0.93
C4—H4	0.93	C17—C18	1.329 (8)
C5—N1	1.373 (5)	C17—H17	0.93
C5—H5	0.93	C18—C19	1.396 (7)
C6—O2	1.257 (5)	C18—H18	0.93
C6—O3	1.257 (5)	C19—H19	0.93
C6—C7	1.502 (6)	N1—Ni1	2.042 (3)
C7—C12	1.388 (6)	N2—H2	0.8600
C7—C8	1.403 (6)	Ni1—N1^i^	2.042 (3)
C8—C9	1.378 (6)	Ni1—O2^i^	2.094 (3)
C8—H8	0.93	Ni1—O2	2.094 (3)
C9—C10	1.361 (7)	Ni1—O1^i^	2.136 (3)
C9—H9	0.93	Ni1—O1	2.136 (3)
C10—C11	1.394 (7)	O1—H1	0.78 (5)
			
O1—C1—C2	112.2 (4)	C14—C13—C12	117.9 (4)
O1—C1—H1A	109.2	C15—C14—C19	119.2 (4)
C2—C1—H1A	109.2	C15—C14—C13	122.4 (4)
O1—C1—H1B	109.2	C19—C14—C13	118.4 (4)
C2—C1—H1B	109.2	C14—C15—C16	120.1 (5)
H1A—C1—H1B	107.9	C14—C15—H15	119.9
C1—C2—H2A	109.5	C16—C15—H15	119.9
C1—C2—H2B	109.5	C15—C16—C17	119.6 (6)
H2A—C2—H2B	109.5	C15—C16—H16	120.2
C1—C2—H2C	109.5	C17—C16—H16	120.2
H2A—C2—H2C	109.5	C18—C17—C16	121.1 (6)
H2B—C2—H2C	109.5	C18—C17—H17	119.5
N1—C3—N2	112.0 (4)	C16—C17—H17	119.5
N1—C3—H3	124.0	C17—C18—C19	120.5 (6)
N2—C3—H3	124.0	C17—C18—H18	119.8
C5—C4—N2	105.8 (4)	C19—C18—H18	119.8
C5—C4—H4	127.1	C18—C19—C14	119.5 (5)
N2—C4—H4	127.1	C18—C19—H19	120.2
C4—C5—N1	109.8 (4)	C14—C19—H19	120.2
C4—C5—H5	125.1	C3—N1—C5	105.0 (4)
N1—C5—H5	125.1	C3—N1—Ni1	125.2 (3)
O2—C6—O3	125.1 (4)	C5—N1—Ni1	129.8 (3)
O2—C6—C7	117.4 (4)	C3—N2—C4	107.4 (4)
O3—C6—C7	117.4 (4)	C3—N2—H2	126.3
C12—C7—C8	118.9 (4)	C4—N2—H2	126.3
C12—C7—C6	122.4 (4)	N1^i^—Ni1—N1	180.0
C8—C7—C6	118.7 (4)	N1^i^—Ni1—O2^i^	89.87 (12)
C9—C8—C7	120.6 (5)	N1—Ni1—O2^i^	90.13 (12)
C9—C8—H8	119.7	N1^i^—Ni1—O2	90.13 (12)
C7—C8—H8	119.7	N1—Ni1—O2	89.87 (12)
C10—C9—C8	120.0 (5)	O2^i^—Ni1—O2	180.0
C10—C9—H9	120.0	N1^i^—Ni1—O1^i^	94.64 (12)
C8—C9—H9	120.0	N1—Ni1—O1^i^	85.36 (12)
C9—C10—C11	120.7 (5)	O2^i^—Ni1—O1^i^	90.65 (11)
C9—C10—H10	119.6	O2—Ni1—O1^i^	89.35 (11)
C11—C10—H10	119.6	N1^i^—Ni1—O1	85.36 (12)
C12—C11—C10	119.6 (5)	N1—Ni1—O1	94.64 (12)
C12—C11—H11	120.2	O2^i^—Ni1—O1	89.35 (11)
C10—C11—H11	120.2	O2—Ni1—O1	90.65 (11)
C11—C12—C7	120.2 (4)	O1^i^—Ni1—O1	180.0
C11—C12—C13	116.6 (4)	C1—O1—Ni1	124.9 (3)
C7—C12—C13	123.1 (4)	C1—O1—H1	112 (4)
O4—C13—C14	121.9 (4)	Ni1—O1—H1	88 (4)
O4—C13—C12	120.0 (4)	C6—O2—Ni1	126.8 (3)
			
N2—C4—C5—N1	0.0 (5)	C17—C18—C19—C14	0.4 (8)
O2—C6—C7—C12	−20.1 (6)	C15—C14—C19—C18	0.6 (7)
O3—C6—C7—C12	160.0 (4)	C13—C14—C19—C18	−178.3 (4)
O2—C6—C7—C8	158.0 (4)	N2—C3—N1—C5	−0.6 (5)
O3—C6—C7—C8	−21.9 (6)	N2—C3—N1—Ni1	176.6 (3)
C12—C7—C8—C9	−1.1 (7)	C4—C5—N1—C3	0.3 (5)
C6—C7—C8—C9	−179.2 (4)	C4—C5—N1—Ni1	−176.7 (3)
C7—C8—C9—C10	1.4 (8)	N1—C3—N2—C4	0.7 (5)
C8—C9—C10—C11	−0.7 (8)	C5—C4—N2—C3	−0.4 (5)
C9—C10—C11—C12	−0.4 (8)	C3—N1—Ni1—O2^i^	−147.8 (3)
C10—C11—C12—C7	0.7 (7)	C5—N1—Ni1—O2^i^	28.7 (4)
C10—C11—C12—C13	−175.6 (5)	C3—N1—Ni1—O2	32.2 (3)
C8—C7—C12—C11	0.0 (6)	C5—N1—Ni1—O2	−151.3 (4)
C6—C7—C12—C11	178.1 (4)	C3—N1—Ni1—O1^i^	−57.2 (3)
C8—C7—C12—C13	176.1 (4)	C5—N1—Ni1—O1^i^	119.3 (4)
C6—C7—C12—C13	−5.9 (6)	C3—N1—Ni1—O1	122.8 (3)
C11—C12—C13—O4	100.0 (5)	C5—N1—Ni1—O1	−60.7 (4)
C7—C12—C13—O4	−76.2 (5)	C2—C1—O1—Ni1	−167.2 (3)
C11—C12—C13—C14	−74.9 (5)	N1^i^—Ni1—O1—C1	−179.8 (4)
C7—C12—C13—C14	108.9 (5)	N1—Ni1—O1—C1	0.2 (4)
O4—C13—C14—C15	−178.2 (4)	O2^i^—Ni1—O1—C1	−89.8 (3)
C12—C13—C14—C15	−3.4 (6)	O2—Ni1—O1—C1	90.2 (3)
O4—C13—C14—C19	0.6 (6)	O3—C6—O2—Ni1	−3.1 (6)
C12—C13—C14—C19	175.4 (4)	C7—C6—O2—Ni1	177.0 (2)
C19—C14—C15—C16	−1.0 (7)	N1^i^—Ni1—O2—C6	−61.3 (3)
C13—C14—C15—C16	177.8 (5)	N1—Ni1—O2—C6	118.7 (3)
C14—C15—C16—C17	0.6 (9)	O1^i^—Ni1—O2—C6	−155.9 (3)
C15—C16—C17—C18	0.4 (9)	O1—Ni1—O2—C6	24.1 (3)
C16—C17—C18—C19	−0.8 (9)		

Symmetry codes: (i) −*x*+1, −*y*+1, −*z*+1.

### Hydrogen-bond geometry (Å, °)

**Table:**

*D*—H···*A*	*D*—H	H···*A*	*D*···*A*	*D*—H···*A*
O1—H1···O3	0.78 (5)	1.86 (5)	2.627 (4)	170 (6)
N2—H2···O3^ii^	0.86	2.02	2.837 (5)	159

Symmetry codes: (ii) *x*, *y*−1, *z*.
